# *In vivo* multi-modal imaging of experimental autoimmune uveoretinitis in transgenic reporter mice reveals the dynamic nature of inflammatory changes during disease progression

**DOI:** 10.1186/s12974-015-0235-6

**Published:** 2015-01-27

**Authors:** Xiangting Chen, Jelena M Kezic, John V Forrester, Gabrielle L Goldberg, Ian P Wicks, Claude C Bernard, Paul G McMenamin

**Affiliations:** Department of Anatomy and Developmental Biology, School of Biomedical Sciences, Faculty of Medicine, Nursing and Health Sciences, Monash University, Clayton, Victoria Australia; Section of Immunology and Infection, Division of Applied Medicine, School of Medicine and Dentistry, Institute of Medical Science, Foresterhill, University of Aberdeen, Scotland, UK; Ocular Immunology Program, Centre for Ophthalmology and Visual Science, The University of Western Australia, Crawley, Western Australia Australia; Centre for Experimental Immunology, Lions Eye Institute, Nedlands, Crawley, Western Australia Australia; Walter and Eliza Hall Institute of Medical Research, Parkville, Victoria Australia; Australian Regenerative Medicine Institute, Monash University, Clayton, Victoria Australia

**Keywords:** Experimental autoimmune uveoretinitis, Reporter mice, Clinical imaging, Retinal inflammation, Microglia, Neutrophils, Dendritic cells

## Abstract

**Background:**

Experimental autoimmune uveoretinitis (EAU) is a widely used experimental animal model of human endogenous posterior uveoretinitis. In the present study, we performed *in vivo* imaging of the retina in transgenic reporter mice to investigate dynamic changes in exogenous inflammatory cells and endogenous immune cells during the disease process.

**Methods:**

Transgenic mice (*C57Bl/6 J Cx*_*3*_*cr1*^*GFP/+*^, *C57Bl/6 N CD11c-eYFP*, and *C57Bl/6 J LysM-eGFP*) were used to visualize the dynamic changes of myeloid-derived cells, putative dendritic cells and neutrophils during EAU. Transgenic mice were monitored with multi-modal fundus imaging camera over five time points following disease induction with the retinal auto-antigen, interphotoreceptor retinoid binding protein (IRBP_1–20_). Disease severity was quantified with both clinical and histopathological grading.

**Results:**

In the normal *C57Bl/6 J Cx*_*3*_*cr1*^*GFP/+*^ mouse Cx_3_cr1-expressing microglia were evenly distributed in the retina. In *C57Bl/6 N CD11c-eYFP* mice clusters of CD11c-expressing cells were noted in the retina and in *C57Bl/6 J LysM-eGFP* mice very low numbers of LysM-expressing neutrophils were observed in the fundus. Following immunization with IRBP_1–20_, fundus examination revealed accumulations of Cx_3_cr1-GFP^+^ myeloid cells, CD11c-eYFP^+^ cells and LysM-eGFP^+^ myelomonocytic cells around the optic nerve head and along retinal vessels as early as day 14 post-immunization. CD11c-eYFP^+^ cells appear to resolve marginally earlier (day 21 post-immunization) than Cx_3_cr1-GFP^+^ and LysM-eGFP^+^ cells. The clinical grading of EAU in transgenic mice correlated closely with histopathological grading.

**Conclusions:**

These results illustrate that *in vivo* fundus imaging of transgenic reporter mice allows direct visualization of various exogenously and endogenously derived leukocyte types during EAU progression. This approach acts as a valuable adjunct to other methods of studying the clinical course of EAU.

**Electronic supplementary material:**

The online version of this article (doi:10.1186/s12974-015-0235-6) contains supplementary material, which is available to authorized users.

## Introduction

Uveitis is the fourth leading cause of blindness in the working age population in developed countries [1,2]. Endogenous posterior uveoretinitis makes up 22% of uveitis cases [3]. The aetiology of non-infectious uveoretinitis is unknown in most cases and has been considered to have an autoimmune basis [4,5]. Well-established animal models of experimental autoimmune uveoretinitis have provided a valuable experimental platform for improving our understanding of the disease pathogenesis and mechanisms of autoimmune uveoretinitis [4-8].

Experimental autoimmune uveoretinitis (EAU) is an organ-specific, T-cell mediated disease that can be induced in susceptible mouse strains by direct immunization with retinal antigens, including interphotoreceptor retinoid binding protein (IRBP) or arrestin (retinal soluble antigen, S-antigen), in complete Freund’s adjuvant and a simultaneous intraperitoneal injection of pertussis toxin. Alternatively, EAU can be induced indirectly by adoptive transfer of retinal antigen-specific effector T cells [4]. Disease usually develops around 10 to 14 days after immunization and is clinically evident within the retina as inflammatory cell infiltration which will include macrophages [9], dendritic cells (DCs) [10], neutrophils [11] and T cells [9,11]. In addition to the influx of blood-derived leukocytes, the resident myeloid-derived macrophages, retinal microglia, are also activated during EAU [7,8].

The Cx_3_cr1^gfp/+^ transgenic knock-in mouse [12] has enabled exquisite *in vivo* and *ex vivo* visualization of the dynamic changes in microglia in steady and diseased states in the eye [13-17], as well as in the brain [18,19], and in particular their rapid responsiveness to injury and presence of noxious stimuli [14,15]. The study of *Cx*_*3*_*cr1*-bearing brain microglia *in vivo* in these reporter mice requires the surgical creation of a defect or window in the calvaria and two-photon microscopic examination of the superficial cortex [20]. By contrast, the eye offers unique advantages for direct *in vivo* visualization of infiltrating and resident immune cells in reporter mice with minimal experimental manipulation [16,21-23]. However, to date there has been limited use of reporter mice to investigate the dynamics of infiltrating leukocytes and resident myeloid cells in an ocular model of autoimmunity.

In the present study, we demonstrate that *in vivo* fundus examination of transgenic reporter mice facilitates monitoring of the dynamic changes in various exogenously and endogenously derived cells of myeloid lineage during EAU progression. In particular we chose to examine EAU in transgenic mice (*C57Bl/6 J Cx*_*3*_*cr1*^*GFP/+*^, *C57Bl/6 CD11c-eYFP*, and *C57Bl/6 J LysM-eGFP*) in which promoter elements of the myeloid-specific *Cx*_*3*_*cr1*, *CD11c* and *lysM* genes are expressed alongside a specific fluorescent reporter in an attempt to characterize the relative temporal pattern of resident and infiltrating myeloid cells, DCs, and neutrophils, respectively. Whilst we appreciate that none of these transgenic reporter mice provide definitive identification of any of the above myeloid cell subsets and thus have their limitations [24], our results do provide novel insights into the cell mediated immune events in this model of human endogenous posterior uveoretinitis and allow accurate clinical grading of disease severity that correlates with histopathological changes.

## Materials and methods

### Mice

Transgenic reporter mice in which the myeloid-specific promoter of the *Cx*_*3*_*cr1*, *CD11c*, and *lysM* genes drives the expression of fluorescent reporters (*C57Bl/6 J Cx*_*3*_*cr1*^*GFP/+*^, *C57Bl/6 N CD11c-eYFP*, and *C57Bl/6 J LysM-eGFP* mice, respectively) were used in this study at an age of between 8 and 10 weeks. The details of the myeloid promoter and reporter mice used in this study are summarized in Table 1. *C57Bl/6 J Cx*_*3*_*cr1*^*GFP/+*^ mice were created by crossing homozygous *C57Bl/6 J Cx*_*3*_*cr1*^*GFP/GFP*^ mice, originally obtained from Professor Steffen Jung [12], to wild-type *C57Bl/6 J* mice. *C57Bl/6 N CD11c-eYFP* mice express enhanced yellow fluorescent protein (eYFP) under the promoter for the *Itgax* (CD11c) gene and are widely regarded as a valuable model for investigating the distribution of DCs with the caveat that other cells are capable of limited expression of CD11c [25]. *C57Bl/6 N CD11c-eYFP* breeding pairs were kindly provided by Associate Professor Michael Hickey (Monash University, Clayton, VIC, Australia) with permission from Professor Michel Nussenzweig (The Rockefeller University, New York, NY, USA) from whom the original mice were obtained [25]. *C57Bl/6 J Cx*_*3*_*cr1*^*GFP/+*^ and *C57Bl/6 CD11c-eYFP* mice were bred at the Monash Large Animal Facility (Monash University). *C57Bl/6 J LysM-eGFP* mice were kindly provided by Associate Professor Michael Hickey. *C57Bl/6 J LysM-eGFP* mice were created by cloning enhanced green fluorescent protein (eGFP) into the lysozyme M (LysM) locus where LysM is expressed in myelomonoytic cells (macrophages and neutrophil granulocytes) [26]. *C57Bl/6 J Cx*_*3*_*cr1*^*GFP/+*^ and *C57Bl/6 J LysM-eGFP* mice were screened by PCR [22] and determined to be negative for retinal degeneration 8 (*rd8*) mutation in the crumbs 1 (*Crb1*) gene (data not shown). *C57Bl/6 N CD11c-eYFP* mice were found to carry the *rd8* mutation and this has been described in detail elsewhere [22]. All experimental animals were maintained under 12:12-hour light/dark cycle with *ad libitum* access to food and water. All procedures in this study were approved by the Monash Animal Ethics Committee (MARP/2011/094) and were performed in accordance with the ARVO Statement for the Use of Animals in Ophthalmic and Vision Research.

**Table 1:** **Transgenic reporter mouse lines**

**Transgenic reporter mouse line**	**Description**	**Specificity**	**Research use**
*C57Bl/6 J Cx*_*3*_ *cr1* ^*gfp/+*^	Knock-in mouse line where one copy of the *Cx*_*3*_ *Cr1* gene is replaced by the GFP reporter gene [12].	Cx_3_Cr1 (GFP^hi^) is expressed in monocytes, subsets of natural killer and T cells, DCs, and microglia.	Extensively used to study monocytes during normal and diseased state in gut [72,73], kidney [74,75], brain [76,77], and the retina of the eye [8,78].
*C57Bl/6 N CD11c-eYFP*	Reporter mouse line that expresses eYFP under the CD11c (*Itgax*) promoter [25].	CD11c is a cell surface molecule expressed on myeloid cells, lymphocytes, natural killer cells and DCs [79]. In *C57Bl/6 N CD11c-eYFP* mice, DCs are characterized by CD11c-eYFP^hi^ while B and T cells are CD11c-eYFP^lo^ [25].	Extensively used to study distribution of DCs in numerous organs including the brain [31,32,80], lung [34], skin [35], gut [36], and the cornea of the eye [33].
*C57Bl/6 J LysM-eGFP*	Reporter mouse line that expresses eGFP under the LysM promoter [26].	LysM is expressed in neutrophil granulocytes (LysM-eGFP^hi^) and macrophages (LysM-eGFP^lo^) [26,37].	Widely used to study neutrophil extravasation during infection [81,82] and inflammation [37,83].

DC, dendritic cell; GFP, green fluorescent protein; eGFP, enhanced green fluorescent protein; eYFP, enhanced yellow fluorescent protein; LysM, Lysozyme M.

### Induction of experimental autoimmune uveoretinitis

EAU was induced using a standard protocol, previously described by others [8]. Briefly mice received a subcutaneous (s.c.) injection of a total of 100 μl emulsion, containing a 50:50 mix of 400 μg human IRBP peptide 1–20 (IRBP_1–20_; GPTHLFQPSLVLDMAKVLLD; China Peptides, Shanghai, China) in complete Freund’s adjuvant (CFA; Sigma-Aldrich, St Louis, MO, USA) supplemented with *Mycobacterium tuberculosis* H37RA (2.5 mg/ml; BD PharMingen, San Diego, CA, USA), distributed between the base of the tail and the right flank of each mouse. Mice simultaneously received an intraperitoneal (i.p.) injection of 1.5 μg pertussis toxin (Sigma-Aldrich) in phosphate-buffered saline (PBS). Control mice received s.c. injection of a total of 100 μl emulsion containing 50:50 mixture of CFA and PBS (no IRBP) and i.p. injection of pertussis toxin.

### Clinical examination of eyes

Mice were clinically examined on day (d) 0, 14, 21, 28 and 35 post-immunization (p.i.). Mice were anesthetized by an i.p. injection of a mixture of ketamine (80 mg/kg; Troy Laboratories, Glendenning, NSW, Australia) and xylazine (10 mg/kg; Troy Laboratories). The pupils were dilated with 0.5% tropicamide (Mydriacil, Alcon Laboratories, Vilvoorde, Belgium), and the cornea was kept moist with the application of sterile lubricant GenTeal gel (Novartis, North Ryde, NSW, Australia). The fundus was examined using the Micron III camera (Phoenix Research Laboratories, Pleasanton, CA, USA) with StreamPix 5 software. Examination consisted firstly of capturing short 5 seconds videos with 100 frames in brightfield, followed by a sequence of GFP^+^ or YFP^+^ cells using the green fluorescent barrier filters, and lastly by a short sequence following a 20 μl s.c. injection of 10% fluorescein isothiocyanate (Alcon Laboratories, Frenchs Forest, NSW, Australia) to visualize the retinal vasculature. The brightness and contrast of all fundus images were adjusted equally using ImageJ 1.48C software [27].

### Clinical assessment of experimental autoimmune uveoretinitis

The severity of disease was graded based on examination of the three modes of clinical fundus images (brightfield, fluorescent, and fluorescein angiography) by two masked observers using a modification of a previously described clinical grading scheme for EAU [6]. The modifications to the grading criteria (Table 2) were primarily a consequence of having both traditional brightfield fundus views augmented by the fluorescent capability offered by the Micron III camera.

**Table 2:** **Experimental autoimmune uveoretinitis clinical grading criteria**

**Score**	**Retinal tissue infiltrate**	**Optic disc**	**Retinal atrophy**	**Retinal detachment**	**Vasculitis**	**Haemorrhages**	**Fluorescein leakage**	**Vitreous haze**
0	<5 small lesions	No inflammation	Absent	Absent	Absent	Absent	Absent	Fundus details clearly visible
1	>5 small linear lesions	Minimal inflammation	Absent	Absent	Minimal thickening	Absent	Mild	Fundus details clearly visible
2	>5 medium linear lesions	Mild inflammation	Absent	Absent	Minimal thickening and localised severe thickening	Absent	Moderate	Fundus details clearly visible
3	>5 large linear lesions	Moderate inflammation	Absent	Absent	Localised severe thickening on all vessels or one vessel grossly affected	Present	Severe	Fundus details visible
4	Confluent large linear lesions	Severe inflammation	Present	Present	One or all vessels grossly affected	Present	Severe	Fundus details slightly unclear
5	Confluent large linear lesions	Severe inflammation	Present	Present	One or all vessels grossly affected	Present	Severe	Fundus details not visible

### Histological assessment of experimental autoimmune uveoretinitis

Mice were euthanized on d35 p.i. with an i.p. injection of sodium pentabarbitone (Lethabarb; Virbac, Milperra, NSW, Australia) and were perfused with 1% heparin in PBS followed by 4% paraformaldehyde (PFA) in PBS. Eyes were enucleated and stored in 4% PFA at 4°C. The right eye from each animal was post-fixed with Karnovsky fixative (4% PFA: 1% glutaraldehyde) for 48 hours and processed for resin histology. Tissues were embedded in Technovit glycol methacrylate resin (Heraeus Kulzer, Wehrheim, Germany), sectioned at 5 μm thickness through the optic nerve-pupillary axis and stained with haematoxylin and eosin. Sections from three different levels of each eye separated by a minimum distance of 50 μm were examined by light microscopy and disease severity was scored by a masked observer using the previously published histopathology grading system for mouse EAU [28].

## Results

### Imaging of experimental autoimmune uveoretinitis disease progression in *C57Bl/6 J Cx*_*3*_*cr1*^*GFP/+*^ mice

**Figure 1:**
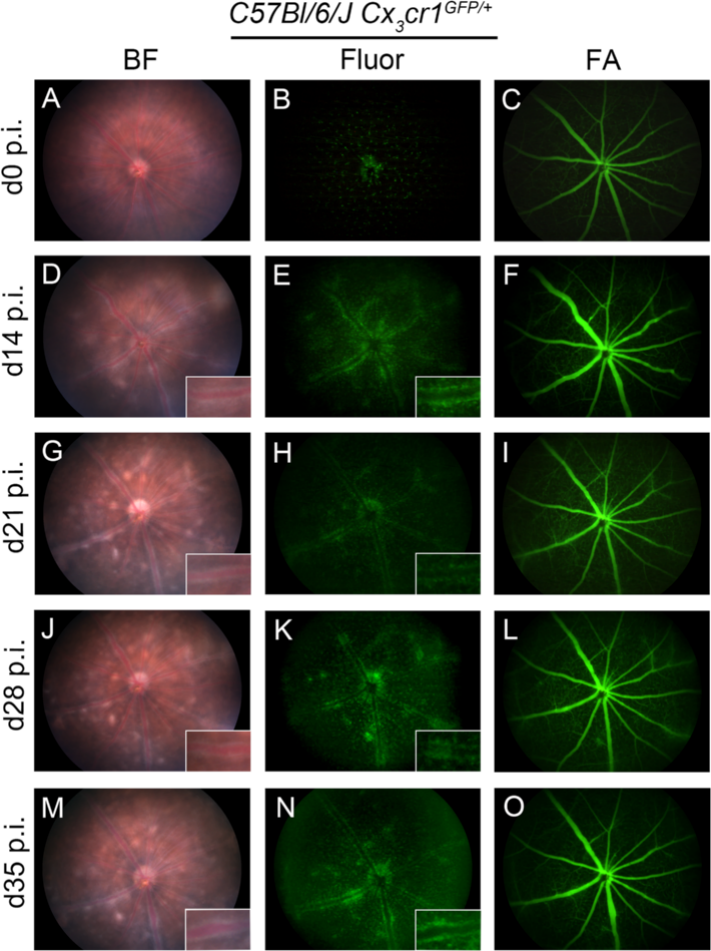
**Time-course monitoring of experimental autoimmune uveoretinitis disease with multi-modal imaging in one representative** ***C57Bl/6 J Cx***_***3***_ ***cr1*** ^***GFP/+***^ **mouse.** Fundus images of a *C57Bl/6 J Cx*_*3*_ *cr1* ^*GFP/+*^ mouse immunized with IRBP_1–20_ in brightfield (BF) **(A,D,G,J,M)**, fluorescent (Fluor) **(B,E,H,K,N)**, and fluorescein angiography (FA) **(C,F,I,L,O)** taken over five time points illustrated experimental autoimmune uveoretinitis disease progression and GFP^+^ myeloid cell perivascular infiltrates during disease progression. d, Day; p.i. post-immunization. GFP, green fluorescent protein; IRBP, interphotoreceptor retinoid binding protein.

*In vivo* clinical examination of eyes in *C57Bl/6 J Cx*_*3*_*cr1*^*GFP/+*^ mice prior to immunization revealed a normal fundus using brightfield ophthalmoscopy (Figure 1A), and revealed the distribution of the retinal microglia network in green fluorescent mode (Figure 1B) and normal retinal vasculature following fluorescein angiography (FA; Figure 1C). The time course of EAU was monitored in *C57Bl/6 J Cx*_*3*_*cr1*^*GFP/+*^ mice immunized with IRBP_1–20_ over four time points (d14, d21, d28, and d35 p.i.). EAU disease was first observed on d14 p.i. (Figure 1D-F), was maximal at d21 p.i. (Figure 1G-I) and d28 p.i. (Figure 1J-L), and was only slightly reduced in severity at d35 p.i. (Figure 1M-O). All IRBP_1–20_ immunized mice developed EAU while adjuvant treated control mice appeared normal at all time points (Additional file 1: Figure S1A-C).

### Clinical examination of *C57Bl/6 J Cx*_*3*_*cr1*^*GFP/+*^ mice during experimental autoimmune uveoretinitis reveals different disease severities

EAU disease severity can vary in IRBP_1–20_ immunized *C57Bl/6 J Cx*_*3*_*cr1*^*GFP/+*^ mice from mild (Additional file 1: Figure S1D-F), moderate (Additional file 1: Figure S1G-I) to severe (Additional file 1: Figure S1J-L) at all time points. Mild disease is characterized by swelling of the optic nerve head and mild vasculitis or perivenular cuffing (Additional file 1: Figure S1D), both evident due to accumulations of Cx_3_cr1-GFP^+^ myeloid cells (Additional file 1: Figure S1E). However, no fluorescein leakage was noted in FA at this point although only the early filling phase was examined (Additional file 1: Figure S1F). Moderate grades are characterised by multiple small focal retinal lesions with moderate vasculitis affecting all retinal vessels evident in brightfield (Additional file 1: Figure S1G) and as Cx_3_cr1-GFP^+^ perivenular infiltrates (Additional file 1: Figure S1H) with an absence of fluorescein leakage (Additional file 1: Figure S1I). Despite the presence of Cx_3_cr1-GFP^+^ perivenular infiltrates, Cx_3_cr1-GFP^+^ cellular infiltrates were absent in some focal retinal lesions seen in brightfield (Additional file 1: Figure S1G). Severe EAU can occasionally be detected as early as d14 p.i. and in the eyes where EAU disease was seen to be severe, multiple large retinal lesions and vasculitis affecting all retinal veins was noted (Additional file 1: Figure S1J). Marked Cx_3_cr1-GPF^+^ peri-vascular infiltrates (Additional file 1: Figure S1K) sometimes correlated with leakage of fluorescein (Additional file 1: Figure S1L). The short 5 second brightfield fundus video also demonstrated rolling and migration of Cx_3_cr1-GFP^+^ cells and non-Cx_3_cr1-GFP^+^ cells in the retinal veins (Additional file 2: Supplementary video 1).

To eliminate the possibility that the hyperfluorescent perivascular infiltrates in *C57Bl/6 J Cx*_*3*_*cr1*^*GFP/+*^ mice are due to autofluorescence inflammatory cells we have provided clinical fundus images of IRBP_1–20_ immunized *Wildtype C57Bl/6 J* taken at d21 p.i. (Additional file 3: Figure S2).

### Clinical grading strongly correlates with histopathological grade of experimental autoimmune uveoretinitis disease in *C57Bl/6 J Cx*_*3*_*cr1*^*GFP/+*^ mice at day 35 post-immunization

**Figure 2:**
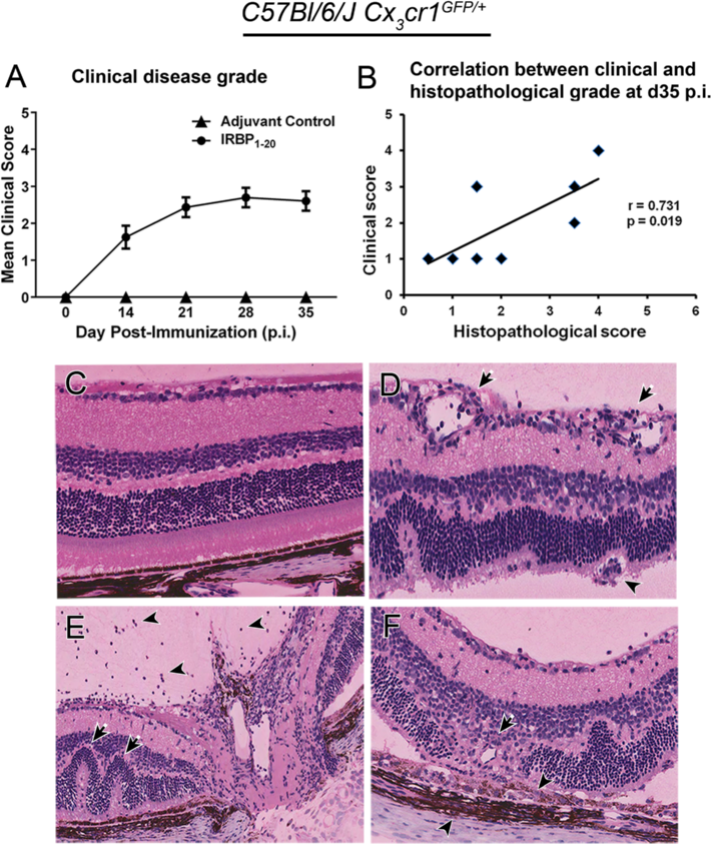
**Comparison of experimental autoimmune uveoretinitis clinical and histopathological score in** ***C57Bl/6 J Cx***_***3***_ ***cr1*** ^***GFP/+***^ **mice.** No adjuvant controls developed experimental autoimmune uveoretinitis (EAU). EAU clinical disease score **(A)** in *C57Bl/6 J Cx*_*3*_ *cr1* ^*GFP/+*^ mice revealed a grade 2.8 disease by day 35 (d35) post-immunization (p.i.) (adjuvant controls: d0, d14, and d28: n = 14, d21 and 35: n = 24; IRBP_1–20_: d0: n = 33, d14: n = 24, d21: n = 32, d28: n = 20, d35: n = 28). **(B)** Correlation between total histopathological disease score in *C57Bl/6 J Cx*_*3*_ *cr1* ^*GFP/+*^ mice on d35 p.i. and the clinical grade in the same mice (IRBP_1–20_: n = 8). Pearson’s correlation was applied to analyse the correlation and the correlation coefficient (r) illustrate a strong positive linear correlation that is significant, *P* < 0.05. **(C)** Histology demonstrated no pathology in adjuvant controls. Representative histology images from *C57Bl/6 J Cx*_*3*_ *cr1* ^*GFP/+*^ mice immunized with IRBP_1-20_ illustrate key histopathological features of EAU including vasculitis (**D**; arrows), granuloma (**D**; arrowhead), retinal folds (**E**; arrows), vitritis (**E**; arrowheads), loss of retinal outer nuclear layer and photoreceptor layer (**F**; arrow), and cellular infiltrates of the retinal pigment epithelium and choroid (**F**; arrowheads). IRBP, interphotoreceptor retinoid binding protein.

EAU clinical disease scores from all clinical imaging time points were determined by two masked observers using the grading criteria (Table 2). All adjuvant controls appeared normal at all time points (Figure 2A) and no histopathological changes were observed at d35 p.i. (Figure 2C). In IRBP_1–20_ immunized *C57Bl/6 J Cx*_*3*_*cr1*^*GFP/+*^ mice EAU began to develop around d14 p.i. with grade 1.86 ± 0.33 (mean ± SEM) disease then gradually progressed over time and remained at grade 2.81 ± 0.24 until d35 p.i. (Figure 2A).

Histopathological disease scores in *C57Bl/6 J Cx*_*3*_*cr1*^*GFP/+*^ mice at d35 p.i. revealed a mean infiltrative grade of 2.38 ± 0.60 and a structural grade of 2.00 ± 0.38. Total histopathological score shows a significant positive correlation (r = 0.73; *P* = 0.019) with the clinical scores (Figure 2B). *C57Bl/6 J Cx*_*3*_*cr1*^*GFP/+*^ mice immunized with IRBP_1–20_ displayed classical histopathological features of EAU (Figure 2D-F), which have been described previously by several authors [6,28-30].

### Clinical examination of experimental autoimmune uveoretinitis in *C57Bl/6 N CD11c-eYFP* mice

**Figure 3:**
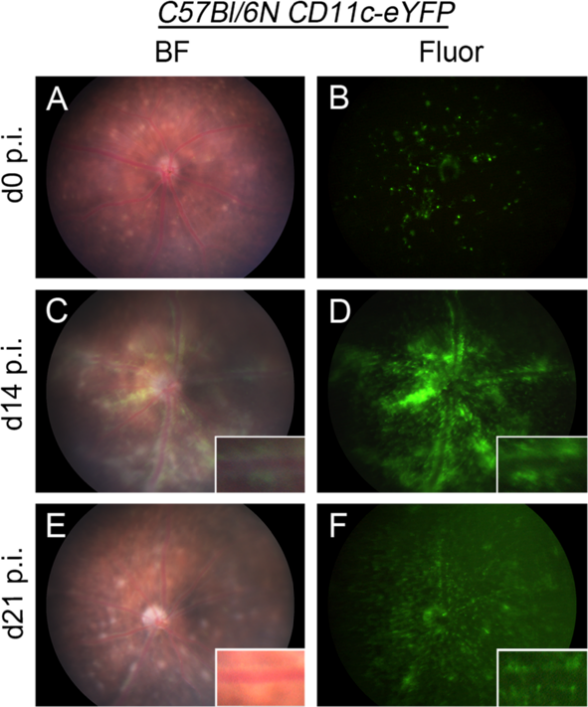
**Clinical fundus examination of** ***C57Bl/6 N CD11c-eYFP*** **mice in normal and diseased state.** Fundus images of naïve *C57Bl/6 N CD11c-eYFP* (*Crb1* ^*rd8*^) mice in brightfield (BF) **(A)** and fluorescent mode (Fluor) **(B)** displaying multiple retinal lesions and accumulation of CD11c-eYFP^+^ cells in the retinal lesions attributable to the *rd8* mutation. Fundus images of *C57Bl/6 N CD11c-eYFP* mice (n = 10) immunized with IRBP_1–20_ on day 14 post-immunization (d14 p.i.) **(C-D)** and d21 p.i. **(E-F)** demonstrated perivascular infiltrates. eYFP, enhanced yellow fluorescent protein; rd8, retinal degeneration 8.

The *C57Bl/6 N CD11c-eYFP* mouse has been extensively used to map putative DCs in a variety of tissues [25,31-36]. We have recently described our discovery of the presence of the *rd8* mutation in the *Crb1* gene in the *C57Bl/6 N CD11c-eYFP* mice [22]. Multiple retinal lesions (Figure 3A) were observed in the fundus of naïve *C57Bl/6 N CD11c-eYFP* mice and we illustrated that these lesions co-localized with CD11c-eYFP^+^ cells (Figure 3B) [22]. At the time of our experiments to investigate the course of EAU in *C57Bl/6 N CD11c-eYFP* mice we were unaware of the *rd8* mutation. It was during clinical examination of d0 naïve animals that we became suspicious of a baseline pathological status. Despite the presence of retinal degeneration, EAU was readily induced and typical EAU changes were noted in *C57Bl/6 N CD11c-eYFP* mice on d14 and d21 p.i. By d14 p.i. severe vasculitis was seen on brightfield fundoscopy (Figure 3C) and a large influx of perivascular CD11c-eYFP^+^ cells along retinal veins was observed in the fluorescent mode (Figure 3D) as well as in the brightfield due to the strong eYFP signal. Clinical examination on d21 p.i. revealed that vasculitis and the large influx of CD11c-eYFP^+^ cells was partly resolved but multiple retinal lesions were still present, and CD11c-eYFP^+^ cells were more evenly distributed in the retina (Figure 3E,F). FA examination revealed absence of fluorescein leakage (data not shown). All IRBP_1–20_ immunized mice developed EAU while adjuvant treated control mice appeared normal at all time points.

### Visualization of LysM-eGFP^+^ myelomonocytic cell infiltration during experimental autoimmune uveoretinitis in *C57Bl/6 J LysM-eGFP* mice

**Figure 4:**
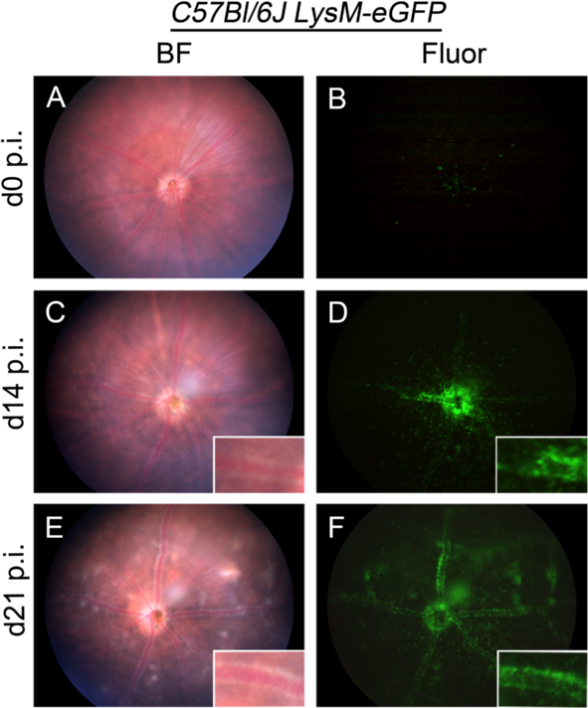
**Clinical fundus examination of** ***C57Bl/6 J LysM-eGFP*** **mice in normal and diseased state.** Fundus images of naïve *C57Bl/6 J LysM-eGFP* mouse fundus in brightfield (BF) **(A)** and fluorescent mode (Fluor) **(B)** appears normal (n =5). Fundus images of *C57Bl/6 J LysM-eGFP* mouse (n = 4) immunized with IRBP_1–20_ on day 14 post-immunization (d14 p.i.) **(C-D)** and d21 p.i. **(E-F)** illustrated the peripapillary infiltrates of vasculitis. eGFP, enhanced green fluorescent protein.

In *C57Bl/6 J LysM-eGFP* mice, eGFP is cloned into the LysM locus which is expressed in myelomonocytic cells (macrophages-eGFP^low^ and neutrophils-eGFP^high^) [26] and have been used for *in vivo* imaging of neutrophils in healthy and diseased states [37]. *In vivo* imaging of the naïve *C57Bl/6 J LysM-eGFP* mouse fundus (n = 5) revealed a normal appearance (Figure 4A) and genotyping of these mice demonstrated that they did not carry the *rd8* mutation (data not shown). In the naïve retina of these mice there were 16.6 ± 1.18 (mean ± SEM) LysM-eGFP^+^ cells present in the entire normal fundus (Figure 4B). However, upon examination of the clinical fundus video, transient LysM-eGFP^+^ cells were observed travelling at high velocity through the lumina of the retinal vessels (Additional file 4: Supplementary video 2). *In vivo* imaging of IRBP_1–20_ immunized *C57Bl/6 J LysM-eGFP* mouse fundus on d14 p.i. illustrated mild EAU with swelling of the optic nerve head, mild vasculitis (Figure 4C) and accumulations of the LysM-eGFP^+^ cells around the optic nerve head and along the retinal vessels (Figure 4D). Clinical fundus examination on d21 p.i. revealed multiple large retinal lesions and moderate vasculitis (Figure 4E) and the LysM-eGFP^+^ cells were found along the retinal vessels and in retinal lesions (Figure 4F). FA examination revealed absence of fluorescein leakage (data not shown). All IRBP_1–20_ immunized mice developed EAU while adjuvant treated control mice appeared normal at all time points.

## Discussion

Intravital imaging using genetically modified reporter mice in which leukocyte subtypes are endogenously labelled with a fluorescent reporter gene transcript has greatly enhanced our understanding of cellular and immunological mechanisms during inflammation of several tissues [38-40]. Many of these experimental approaches are partly hindered or complicated by the potential effects of surgical intervention needed to exteriorise or surgically alter the tissue under investigation such as mesentery [41,42], cremaster muscle [42-44], liver [45], lung [37], kidney [40,43], and skin [46]. In the case of intravital imaging of the brain a craniotomy window is required [47,48]. The eye has several advantages over most organs because by its very nature it provides a clear transparent window on both neural tissue (retina) and connective tissues (cornea, iris) [33,49], thus avoiding surgical intervention. We sought to exploit the recent development of multi-modal imaging techniques which allow high-quality examination of the mouse fundus [16,22] to investigate the behaviour of cells of myeloid origin during the course of EAU, a widely used model of ocular autoimmune disease.

Disease severity of EAU is routinely determined using histopathological grading methods [28,29,50]. To circumvent the issue of single time point post-mortem grading, several research groups have developed non-invasive clinical grading methods including topical endoscopic fundus imaging [51], otoscope imaging [6], scanning laser ophthalmoscopy [52] and optical coherence tomography [53-55]. In this present study, multi-modal fundus ophthalmoscopy was chosen to grade the disease severity as it has the advantages of being relatively inexpensive and has the capability of capturing both video and still frame images in brightfield, together with green and red fluorescence wavelengths.

Many previous phenotypic analyses of the inflammatory cell infiltrate during EAU have used multi-parameter flow cytometry to show that the majority of infiltrating cells are myeloid-derived with a peak in T cell infiltration around d14 p.i. [11,56,57]. Alternative approaches to visualize leukocyte trafficking *in vivo* in the eye during EAU have included use of acridine orange, a non-specific nuclear dye which can be visualized by fluorography. This method revealed leukocytes rolling along the retinal veins as early as d14 p.i. [58,59]; however, their specific phenotype was obviously not determined. More recently, *in vivo* imaging of the leukocyte subtypes with more specificity has become easier with the availability of genetically modified mouse models in which genes regulating leukocyte subtypes are used as promoters to express fluorescent reporter proteins. Although these transgenic mouse models are useful for providing insights to myeloid lineage cell types in normal and diseased state, some authors have warned of cautious interpretation of these mice as the sole means of identifying and distinguishing macrophages and DCs [24]. Such limitations are also true of the three transgenic mouse lines chosen for the present study.

There are several subpopulations of Cx_3_cr1-GFP^+^ myeloid-derived cells in the normal retina including the hyalocytes on the retinal surface [60], subretinal macrophages on the other aspect of the neural retinal [61-63], and the extensive network of microglial populations in the retinal parenchyma [13,64]. *C57Bl/6 J Cx*_*3*_*cr1*^*GFP/+*^ mice [12] have been widely used to investigate the role of microglia in numerous ocular conditions that may have an inflammatory element in their pathogenesis including potential models of retinal degeneration [65], retinopathy of prematurity [66,67] and diabetic retinopathy [16]. In the present study we demonstrate highly distinctive perivenular infiltrates of Cx_3_cr1-GFP^+^ cells at d14 p.i. to d35 p.i. which is in agreement with previous studies [8,68]. The perivenular infiltrate could theoretically represent haematogenous Cx_3_cr1-GPF^+^ (GFP^low^) myeloid cells recently extravasated into the retina or the chemotactic migration of resident Cx_3_cr1-GPF^+^ (GFP^high^) microglia towards the vasculature. We believe the former is the case as the Cx_3_cr1-GPF^+^ (GFP^high^) microglia network seemed largely undisturbed, something that was subsequently confirmed by retinal whole mount analysis (data not shown).

The difficulty in distinguishing subpopulations of macrophages from cells of DC lineage was the motivation for the creation of CD11c transgenic reporter mice, specifically the *CD11c-eYFP* [25] and *CD11c-DTR/GFP* mice [69]. In these transgenic mice the promoter for the *Itgax* (CD11c) gene is used to drive eYFP expression (*CD11c-eYFP* mice) or GFP and diphtheria toxin receptor (DTR) expression (*CD11c-DTR/GFP* mice). CD11c is a leukocyte integrin comprised of an alpha X subunit that along with CD18, a leukocyte beta 2 integrin polypeptide, forms the CD11c/CD18 heterodimer which is important in leukocyte adhesion, migration and cell to cell interaction during immune responses. CD11c is expressed heterogeneously by different populations of DCs [25] and is important in T cell priming [69]. However, it is also expressed to at least one log lower than DCs on other immune cells such as natural killer cells, subpopulations of macrophages and activated T cells [25]. As such immune cells are not normally a feature of the resting central nervous system, we, like other previous investigators [31,32,70], thought is reasonably safe to assume that in the resting and disease state CD11c-eYFP^+^ cells may represent predominantly DCs. The view that *CD11c-eYFP* mice are valuable for examining the distribution of DCs has recently been strongly challenged by Hume [24] who points out that CD11c has no function in antigen presentation; not all DCs are CD11c^+^ and that not all CD11c^+^ cells are antigen-presenting cells. It was thus with caution that we chose to take advantage of the transgenic *C57Bl/6 N CD11c-eYFP* mice to examine the dynamic of DCs in EAU. Indeed at the commencement of this study we had the further complication of discovering that these mice had a pre-existing retinal dystrophy due to the presence of the *rd8* mutation in the *crb1* gene [71] and that the CD11c-eYFP^+^ cells in the retina represented activated microglia [22]. In the present study, CD11c-eYFP^+^ cells were recruited into the eye at d14 and d21 p.i. in a similar pattern to that observed in *C57Bl/6 J Cx*_*3*_*cr1*^*GFP/+*^ mice leading us to conclude that these CD11c-eYFP^+^ cells are likely a mixture of myeloid-derived cells.

Interestingly, despite the *C57Bl/6 N CD11c-eYFP* mice carrying the *rd8* mutation and the pre-existing disrupted retinal architecture prior to immunization, they did not develop a more severe form of EAU as may have been predicted if one were to assume that this dystrophic condition compromised the immune status of the retina as we have previously concluded [22], although we have not specifically proven that the blood-ocular barrier was compromised.

In the *C57Bl/6 J LysM-eGFP* mouse line generated by Faust and colleagues [26], homologous recombination was used to insert the eGFP gene into the LysM locus. This was chosen because LysM is expressed specifically in the myelomonocytic cell lineage (macrophages and neutrophil granulocytes). Characterization of LysM-eGFP^+^ cells in the blood revealed that the eGFP^high^ polymorphonuclear granulocytes outnumbered LysM-eGFP^+^ monocytes by 50:1. In the present study, we observed exceedingly small numbers (16.6 ± 1.18) of largely static LysM-eGFP^+^ myelomonocytic cells around the optic nerve head in the normal *C57Bl/6 J LysM-eGFP* fundus. We propose that these are likely of monocyte lineage as it is highly unusual to detect extravasated neutophils in the normal retina. However, video analysis (not shown) revealed many LysM-eGFP^+^ travelling at high velocity in retinal vessel lumina, which we conclude are likely to be circulating neutrophils. In these mice we demonstrated increased numbers of LysM-eGFP^+^ cells in the peripapillary retinal vessels at d14 to d21 of EAU. Closer examination of the high power images (see inset, Figure 4) suggests that many of these are marginating in vessel lumina, a pattern which differs from the perivascular infiltrates observed in the *C57Bl/6 J Cx*_*3*_*cr1*^*GFP/+*^ and *C57Bl/6 N CD11c-eYFP* mice. Subsequent flow cytometry of retinal tissue during EAU in *C57Bl/6 J LysM-eGFP* mice revealed these cells to be largely a neutrophilic infiltrate (Goldberg and colleagues, unpublished data).

## Conclusions

In conclusion, *in vivo* fundus imaging of *C57Bl/6 J Cx*_*3*_*cr1*^*GFP/+*^ and *C57Bl/6 N CD11c-eYFP* mice revealed the dynamics of the myeloid cell infiltrates in EAU, particularly the perivenular accumulations. In contrast, the *C57Bl/6 J LysM-eGFP* mice revealed intravascular margination of LysM-eGFP^hi^ neutrophils as well as less distinctive perivascular infiltrates. The grading of disease using *in vivo* imaging of these genetically modified reporter mice correlated strongly with the histopathological changes.

## Additional files

Additional file 1: Figure S1. Clinical appearance of *C57Bl/6 J Cx*_*3*_ *cr1* ^*GFP/+*^ mouse fundus with different severities of EAU. Fundus images of adjuvant control *C57Bl/6 J Cx*_*3*_ *cr1* ^*GFP/+*^ mouse in brightfield (A), fluorescent mode (B), and fluorescein angiography (C) revealed normal retinal vasculature with no fluorescein leakage and normal retinal microglia network. Multi-modal fundus images from *C57Bl/6 J Cx*_*3*_ *cr1* ^*GFP/+*^ mice immunized with IRBP_1–20_ displaying mild (D-F), moderate EAU (G-I), and severe (J-L) EAU. The classical features of EAU include perivascular vasculitis (D and J, arrows) that is associated with GFP^+^ perivascular infiltrates (E and K, arrows) and multiple focal retinal lesions (G, arrows), venular dilation (L, arrows), and fluorescein leakage (L, arrowhead). Additional file 2: Supplementary Video 1. Video of real time *in vivo* visualization of rolling leukocytes in retinal veins of *C57Bl/6 J Cx*_*3*_ *cr1* ^*GFP/+*^ mouse during EAU. Duration: 5 seconds (20 frames per second). Additional file 3: Figure S2. Clinical appearance of immunized with IRBP_1–20_ *WT C57Bl/6 J* mouse fundus on d21 p.i. These fundus images of WT controls in brightfield (A), fluorescent mode (B), and fluorescein angiography (C) revealed that the inflammatory infiltrated did not autofluoresce. Additional file 4: Supplementary Video 2. Video of real time *in vivo* visualization of naïve *C57Bl/6 J LysM-eGFP* mouse fundus revealed static and circulating LysM-eGFP^+^ myelomonocytic cells. Duration: 5 seconds (20 frames per second).

## Abbreviations

CFA: complete Freund’s adjuvant
Crb1: crumbs 1
d: day
DC: dendritic cell
DTR: diphtheria toxin receptor
EAU: experimental autoimmune uveoretinitis
eGFP: enhanced green fluorescent protein
eYFP: enhanced yellow fluorescent protein
FA: fluorescein angiography
i.p.: intraperitoneal
IRBP: interphotoreceptor retinoid binding protein
LysM: lysozyme M
PBS: phosphate-buffered saline
PCR: polymerase chain reaction
PFA: paraformaldehyde
p.i.: post-immunization
rd8: retinal degeneration 8
s.c.: subcutaneous
