# Supplementary figures and images for: In vivo multi-modal imaging of experimental autoimmune uveoretinitis in transgenic reporter mice reveals the dynamic nature of inflammatory changes during disease progression
Source: J Neuroinflammation. 2015 Jan 27;12:17. doi: 10.1186/s12974-015-0235-6 (PMC4336748; doi:10.1186/s12974-015-0235-6)

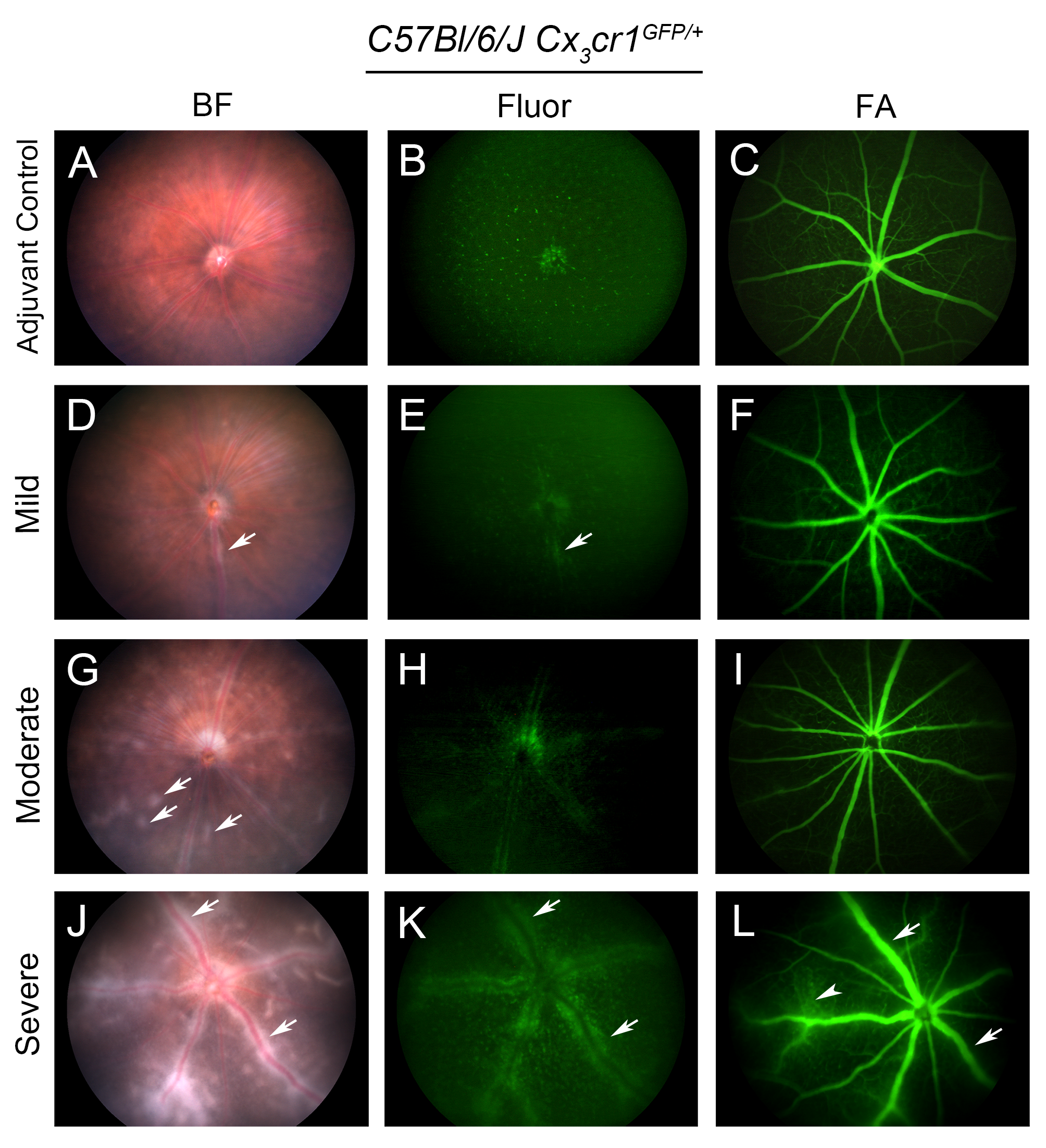

Supplement: Additional file 1: Figure S1. — Clinical appearance of C57Bl/6 J Cx 3 cr1 GFP/+ mouse fundus with different severities of EAU. Fundus images of adjuvant control C57Bl/6 J Cx 3 cr1 GFP/+ mouse in brightfield (A), fluorescent mode (B), and fluorescein angiography (C) revealed normal retinal vasculature with no fluorescein leakage and normal retinal microglia network. Multi-modal fundus images from C57Bl/6 J Cx 3 cr1 GFP/+ mice immunized with IRBP1–20 displaying mild (D-F), moderate EAU (G-I), and severe (J-L) EAU. The classical features of EAU include perivascular vasculitis (D and J, arrows) that is associated with GFP+ perivascular infiltrates (E and K, arrows) and multiple focal retinal lesions (G, arrows), venular dilation (L, arrows), and fluorescein leakage (L, arrowhead). [file 12974_2015_235_MOESM1_ESM.tiff]

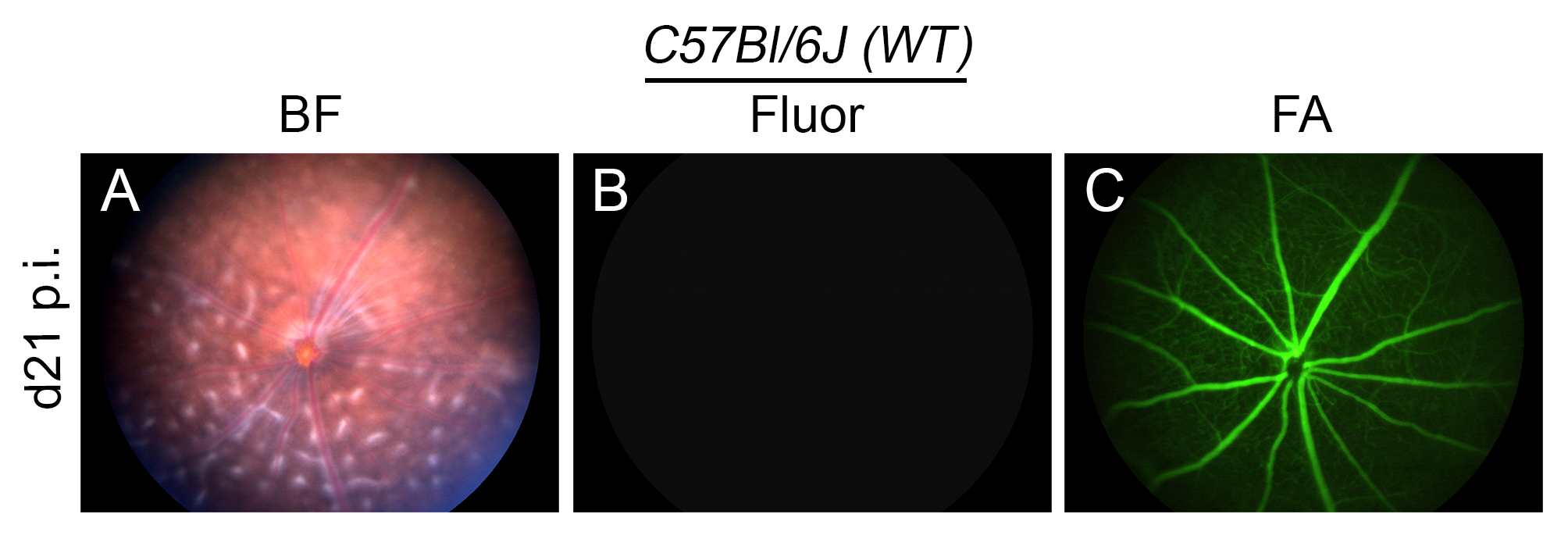

Supplement: Additional file 3: Figure S2. — Clinical appearance of immunized with IRBP1–20 WT C57Bl/6 J mouse fundus on d21 p.i. These fundus images of WT controls in brightfield (A), fluorescent mode (B), and fluorescein angiography (C) revealed that the inflammatory infiltrated did not autofluoresce. [file 12974_2015_235_MOESM3_ESM.tiff]
